# Geoelectrical resistivity data set for characterising crystalline basement aquifers in Basiri, Ado-Ekiti, southwestern Nigeria

**DOI:** 10.1016/j.dib.2018.05.091

**Published:** 2018-05-23

**Authors:** Ahzegbobor P. Aizebeokhai, Olubukola Ogungbade, Kehinde D. Oyeyemi

**Affiliations:** Applied Geophysics Group, College of Science and Technology, Covenant University, Ota, Nigeria

## Abstract

This article consists of data sets for thirty (30) vertical electrical sounding (VES) and four (4) traverses of 2D electrical resistivity imaging (ERI) collected within, Ado-Ekiti, southwestern Nigeria using an ABEM Terrameter (SAS 1000/4000) system. Win-Resist computer program was used to process the apparent resistivity data sets for the VES to determine the geoelectric layers and their respective parameters (resistivity and thickness). The observed data sets for the 2D ERI were processed using RES2DINV software to obtain 2D inverse model resistivity distribution of the subsurface. The resistivity soundings and the 2D ERI were combined to delineate and characterise the crystalline basement features associated with basement aquifers.

**Specifications Table**

**Table:** 

Subject area	Geophysics
More specific ;subject area	Geoelectrical Resistivity
Type of data	Table, Figure, Text file, DAT, RST, and INV files
How data was acquired	Geoelectrical Resistivity Survey using ABEM Terrameter (SAS1000/4000) system.
Data format	Raw, Processed
Experimental factors	The observed apparent resistivity data sets were processed so as to delineate and characterise basement features associated with basement aquifers.
Experimental features	Geophysical survey involving vertical electrical sounding (VES) and 2D electrical resistivity imaging (ERI) was conducted.
Data source location	Ado-Ekiti is between latitude 7°33′−7°42′Nand longitude 5°11′−3°20′E in the crystalline basement complex, southwestern Nigeria.
Data accessibility	All the data sets are with this article.

**Value of the data**

• The geoelectrical resistivity datasets can be used for subsurface characterisation, determination of lithologic layers and delineation of crystalline basement features that are of environmental, geotechnical and hydrogeological/hydrological importance.
• The datasets can be used for geoelectrical characterisation of the weathering profile, and delineation of regolith thickness and fractured and weathered zones which are useful in groundwater potential studies as wells as foundation and geotechnical investigations in crystalline basement complex terrain.
• The data sets can be used for the determination of the spatial variability of basement aquifers as well as zones of significant degree of weathering and fracturing which are areas of preferential accumulation of groundwater; these are useful for siting boreholes and wells in groundwater resource development basement terrain [e.g. [1], [2]].
• The geoelectrical resistivity data sets can be integrated with other geophysical data sets such as induced polarization, magnetic, electromagnetic, ground penetrating radar, gravity and seismic data for detail subsurface characterisation.
• The data set can be used for educational purposes, and for future research in hydrogeological, environmental and geotechnical studies. Similar data articles can be found be found in Refs. [3], [4], [5], [6].
• The data can be compared with those obtained from similar geologic environment.

## 1 Data

The attached files (Appendices A and B) consist of geoelectrical resistivity data sets (vertical electrical soundings (VES) and 2D electrical resistivity imaging (ERI)) used for the delineation and characterisation crystalline basement aquifers. The raw data sets are presented in “dot DAT’ format (DAT files) for both VES and 2D ERI surveys. The processed VES data sets are presented in ‘dot RST’ format; the processed 2D ERI data are presented in ‘dot INV’ format.

## 2 Experimental design, materials and methods

### 2.1 Study area

**Fig. 1:**
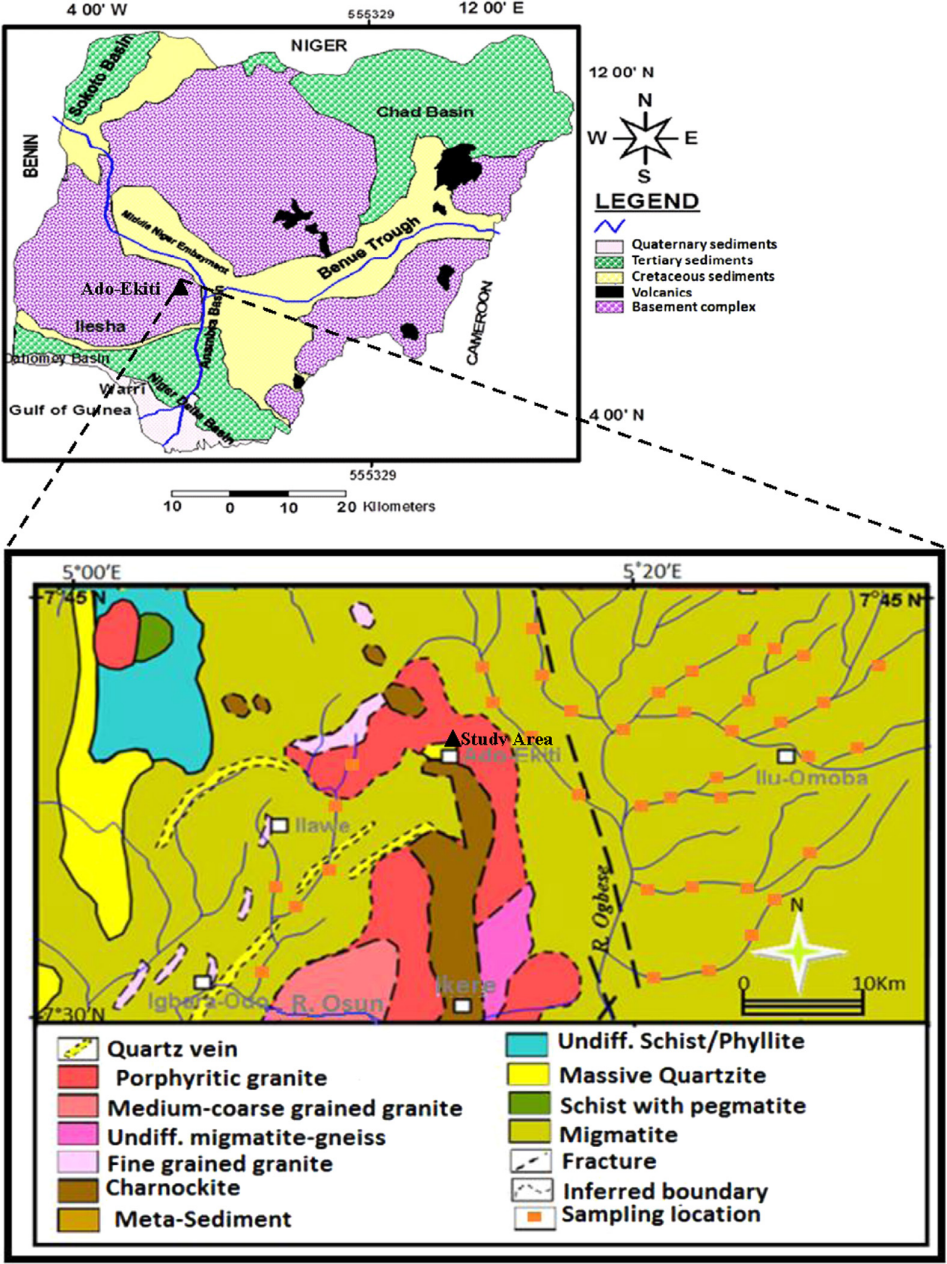
Geological map of Ado-Ekiti and environs showing the location of the study area.

The study area is located Basiri, Ado-Ekiti, Ekiti State, southwestern Nigeria; Ado-Ekiti lies between latitude7°33′−7°42′N and longitude 5°11′−3°20′E. The topography is gentle sloping lowland with several sparsely distributed hills and knolls; mean elevation is about 440 m above mean sea level. The natural vegetation is tropical rain forest. The climate is tropical humid marked by distinct dry and rainy seasons. Precipitation is generally heavy rainfall which distinguishes the climatic seasons. Annual mean rainfall is greater than 2300 mm and forms the main sources of groundwater recharge in the area; monthly temperature ranges from 23 °C in July to 32 °C in February. The area is mainly drained by Rivers Ireje, Elemi, Omisanjana and Awedele which generally flow parallel to the strike of the basement rocks as the rivers and streams are structurally controlled. The area is underlain by crystalline basement rocks of Precambrian age, which are mainly granitic intrusions and highly deformed metamorphic rocks [7], [8], [9]. The dominant rocks are pegmatite, quartz and quartz-schists, biotite granite and undifferentiated gneiss complex (Schist). The weathering of these rocks commonly results in a thick lateritic overburden. Fig. 1 shows the location and geological map of the study area.

### 2.2 Data acquisition

**Fig. 2:**
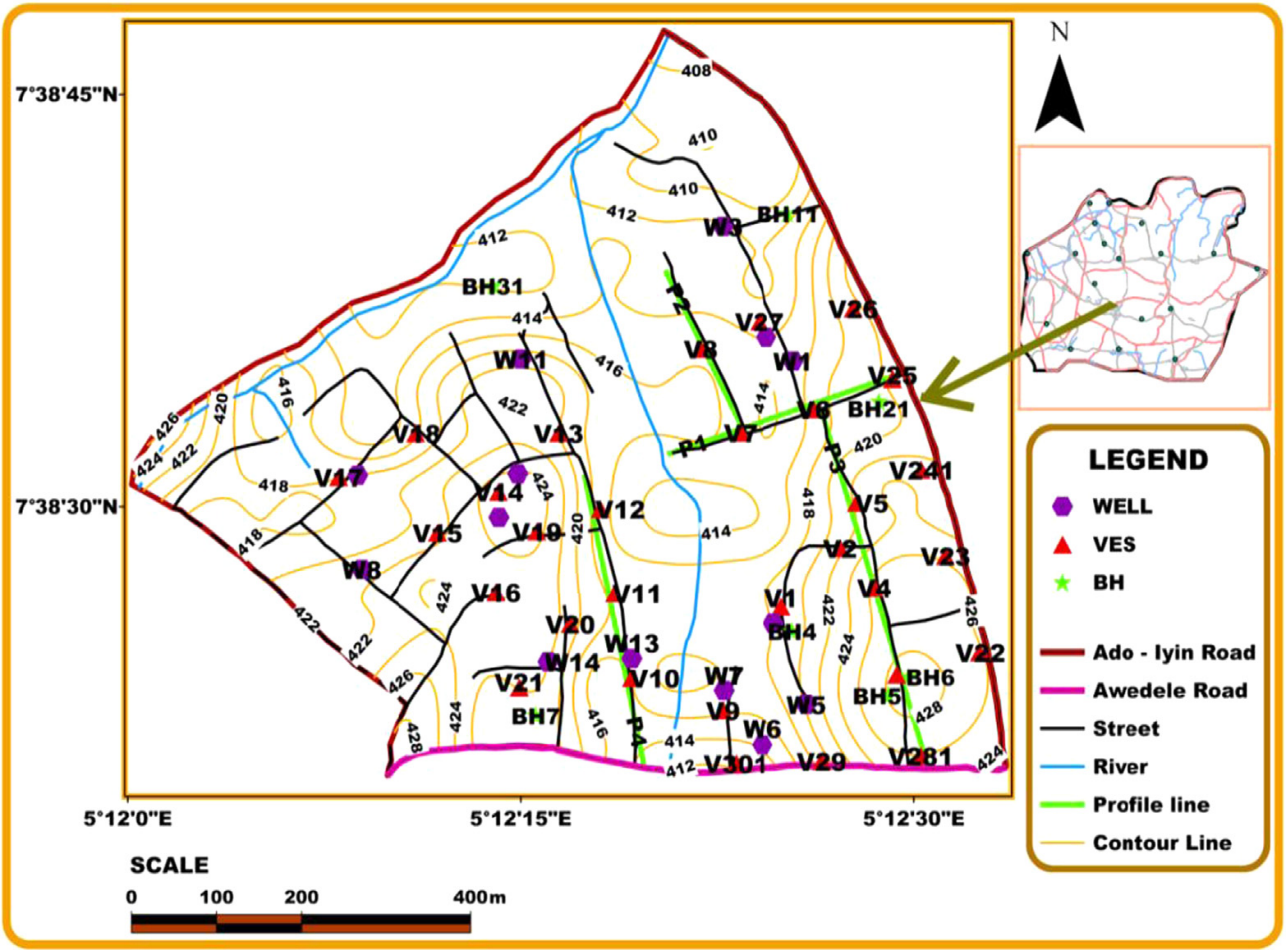
Base map of the study area indicating topography, VES points, 2D traverses and borehole points.

The geoelectrical resistivity survey consists of VES and 2D ERI; the data sets were measured with ABEM Terrameter (SAS1000/4000) system during the onset of the rainy season (April, 2016). The base map showing the locations and distribution of the VES points and 2D ERI traverses is shown in Fig. 2. A total of thirty (30) VESs were conducted across the study area using Schlumberger array with maximum half-current electrode spacing (AB/2) of 100 m and 130 m. The VES survey was conducted so as to determine the subsurface lithologic layering and depth-to-basement at various points in the study area. The GPS coordinates and surface elevation for the VESs points are presented in Table 1. The 2D ERI survey was conducted along four (4) traverses using dipole-dipole array which is sensitive to vertical features such as faults, fractures and dykes of hydrogeological importance in basement terrain [10], [11], [12], [13], [14]; dipole separation factor ranging from 1–4 was used for the 2D survey. Traverse 1 was conducted in the west–east direction while Traverses 2 to 4 were in the south–north direction parallel to the main stream (Awedele stream) that drained the study area (Fig. 2). The profile length of Traverse 1 is 280 m; a dipole length ranging from 5 to 30 m in an interval of 5 m was used for the data measurements. Traverse 2 is about 55 m away from Awedele stream and is 200 m in length; the dipole length for the data measurements ranges from 5 to 65 m in an interval of 5 m. The profile length of Traverse 3 is 420 m and dipole length from 10 to 60 m in an interval of 10 m was used for the measurements. The profile length of Traverse 4 is 320 m and the dipole length used for the measurements ranges from 10 to 40 m in an interval of 10 m. Field techniques for geoelectrical resistivity survey have been discussed in several articles [e.g. [15], [16], [17], [18], [19]].

**Table 1:** GPS coordinates and elevations of the VES points.

**VES**	VES1	VES2	VES3	VES4	VES5	VES6	VES7	VES8	VES9	VES10
**Easting**	5.20693	5.20534	5.20516	5.20501	5.204566	5.203932	5.20328	5.20390	5.20223	5.20306
**Northing**	7.64066	7.63994	7.64079	7.64165	7.642412	7.641819	7.64140	7.64080	7.64197	7.64241
**Elev. (m)**	421.0	424.0	441.0	375.0	412.0	412.0	418.0	413.0	421.0	433.0
**VES**	VES11	VES12	VES13	VES14	VES15	VES16	VES17	VES18	VES19	VES20
**Easting**	5.20434	5.20755	5.20469	5.20452	5.209027	5.208667	5.20841	5.20811	5.20769	5.20669
**Northing**	7.64142	7.64125	7.64048	7.63984	7.640196	7.641171	7.64204	7.64297	7.64367	7.64354
**Elev. (m)**	428.0	398.0	416.0	425.0	421.0	421.0	421.0	423.0	421.0	428.0
**VES**	VES21	VES22	VES23	VES24	VES25	VES26	VES27	VES28	VES29	VES30
**Easting**	5.20841	5.20736	5.20815	5.20644	5.207913	5.207727	5.20727	5.20649	5.20607	5.20630
**Northing**	7.63915	7.63909	7.63997	7.63907	7.640849	7.641709	7.64242	7.64242	7.64326	7.63961
**Elev. (m)**	419.0	408.0	403.0	419.0	419.0	424.0	424.0	411.0	404.0	413.0

### 2.3 Data processing

The observed apparent resistivity data sets for the VES were used to generate field curves which were curve-matched with theoretical master curves for Schlumberger array to estimate the geoelectric layers and their corresponding resistivity and thickness. The estimated geoelectric parameters were used as initial models for computer iteration on Win-Resist computer program to obtain model geoelectric parameters for the delineated layers. The delineated layers and their corresponding geoelectric parameters are shown in Table 2. Similarly, the 2D ERI data sets were processed and inverted using RES2DINV inversion code [11], [18]; the code uses a non-linear optimization technique for determining inverse model of the 2D resistivity distribution. Least-squares inversion technique with standard least-squares constraint (L_2_-norm), which minimizes the square of the difference between the observed and the computed apparent resistivity data set through an iterative procedure, was used for the 2D data inversion. The least-squares equation for the inversion was solved using the standard Gauss-Newton optimization technique and appropriate damping factor was selected based on the estimated noise level on the measured data for each traverse.

**Table 2:** Summary of the geoelectrical parameters obtained from the resistivity soundings.

Layer	1			2			3			4			
Lithology	Top soil/collapsed zone			Upper saprolite (clayey unit)			Lower saprolite (less clayey unit)			Weathered/fractured basement (saprock)			Curve Type
Location	Resistivity (Ωm)	Thickness (m)	Bottom Depth (m)	Resistivity (Ωm)	Thickness (m)	Bottom Depth (m)	Resistivity (Ωm)	Thickness (m)	Bottom Depth (m)	Resistivity (Ωm)	Thickness (m)	Bottom Depth (m)	
VES 1	92.8	1.4	1.4	59.3	5.8	7.2	17.4	11.7	18.9	788.8			QH
VES 2	239.7	0.9	0.9	75.5	10.7	11.6	34.9	20.5	32.1	642.9			QH
VES 3	213.5	0.9	0.9	60.3	5.8	6.7	28.7	14.1	20.8	512.0			QH
VES 4	–	–	–	42.4	17.7	17.7	122.4	15.4	33.1	887.4			A
VES 5	122.2	6.5	6.5	41.1	12.0	18.5	328.5	8.4	26.9	780.9			HA
VES 6	101.8	8.0	8.0	15.4	10.2	18.2	450.7	6.0	24.2	973.7			HA
VES 7	68.8	4.8	4.8	14.4	7.6	12.4	119.6	10.1	22.5	1119.2			HA
VES 8	53.2	4.9	4.9	17.0	12.9	17.8	336.1	8.0	25.8	830.4			HA
VES 9	55.7	3.8	3.8	15.0	14.7	18.5	199.5	–	–	–			H
VES 10	141.9	9.0	9.0	36.8	19.9	38.9	163.8	–	–	–			H
VES 11	161.6	3.3	3.3	342.5	6.4	9.6	28.1	14.0	23.6	325.5			KH
VES 12	136.0	4.3	4.3	408.4	6.0	10.3	37.0	11.0	21.3	346.7			KH
VES 13	61.1	2.0	2.0	197.4	5.3	7.4	67.6	10.9	18.3	260.6			KH
VES 14	190.5	5.4	5.4	389.1	6.9	12.2	37.7	10.0	22.2	716.9			KH
VES 15	128.6	5.6	5.6	135.1	9.9	15.6	262.0	–	–	–			A
VES 16	78.8	6.2	6.2	329.5	8.4	14.6	674.2	–	–	–			A
VES 17	49.7	1.0	1.0	227.4	2.5	3.4	27.7	7.9	11.3	3180.1			KH
VES 18	203.1	2.9	2.9	79.4	9.0	11.9	217.0	9.3	21.2	882.0			HA
VES 19	107.8	6.9	6.9	193.4	8.8	15.7	24.3	14.5	30.2	257.5			KH
VES 20	67.5	2.7	2.7	247.4	5.2	7.8	77.3	6.7	14.6	364.4			KH
VES 21	74.9	11.0	11.0	175.2	7.0	18.0	22.3	–	–	–			K
VES 22	137.3	1.4	1.4	10.2	3.3	4.7	430.2	5.0	9.8	4476.5			HA
VES 23	155.9	2.2	2.2	42.6	11.0	13.2	188.5	–	–	–			H
VES 24	129.6	1.6	1.6	109.9	11.2	12.8	39.4	12.2	29.9	657.8			QH
VES 25	135.9	3.1	3.1	80.0	4.4	7.5	27.6	7.4	11.9	1012.2			QH
VES 26	86.5	6.0	6.0	20.5	18.0	24.0	255.2	–	–	–			H
VES 27	28.2	0.8	0.8	208.0	2.1	2.9	12.3	9.0	12.0	353.9			KH
VES 28	51.8	5.3	5.3	126.1	4.1	9.7	11.6	20.1	29.8	368.5			KH
VES 29	134.7	3.8	3.8	84.3	3.2	7.0	12.8	15.1	22.1	530.8			QH
VES 30	58.7	2.7	2.7	7.3	8.7	11.4	92.6	4.8	16.2	1299.2			HA
